# Finnish experiences of health monitoring: local, regional, and national data sources for policy evaluation

**DOI:** 10.3402/gha.v9.28824

**Published:** 2016-02-29

**Authors:** Katri Kilpeläinen, Suvi Parikka, Päivikki Koponen, Seppo Koskinen, Tuulia Rotko, Timo Koskela, Mika Gissler

**Affiliations:** 1 Department of Welfare, National Institute for Health and Welfare, Helsinki, Finland; 2 Department of Health, National Institute for Health and Welfare, Helsinki, Finland; 3 Department of Information Services, National Institute for Health and Welfare, Helsinki, Finland

**Keywords:** health monitoring, health determinants, Finland

## Abstract

**Background:**

Finland has a long tradition of gathering information about the health and welfare of the adult population.

**Design:**

Surveys and administrative registers form the basis for national and local health monitoring in Finland.

**Results:**

Different data sources are used in Finland to develop key indicators, which can be used to evaluate how the national health policy targets have been met in different parts of the country and in different population subgroups. Progress has been shown in chronic disease risk factors, such as smoking reduction. However, some health policy targets have not been met. Socioeconomic health differences, for example, have remained large compared with other European countries.

**Conclusion:**

Although data availability for key health indicators is good in Finland, there is a need for wider and more comprehensive use of this information by political decision-makers and healthcare professionals.

## Introduction

Over the last few decades, Finnish national health policy has relied on the Health in All Policies (HiAP) approach. *HiAP* refers to a strategy where the impact on the health of the population and various population groups is consciously taken into account by different sectors in their decision-making. The core aim of HiAP is to improve public health by impacting broadly on those determinants of health in which the health sector has limited influence (1). Further, national policy promotes social inclusion to strengthen the health and welfare of specific population groups, including migrants and other minorities (2). As part of this endeavor, local authorities play a crucial role, as the municipalities are responsible for promoting residents’ health and welfare. The legislation also obligates municipalities to recognize health in all their policies and utilize health impact assessment (HIA) to cooperate with other public and private bodies and non-governmental organizations in health promotion, to monitor health and health determinants, to prepare regular welfare reports, and to pay special attention to health inequalities.

In principle, Finland offers universal access to health care to all residents. Until now, primary care has been organized in public municipal health centers and specialist care in 20 hospital districts (3). The private sector also provides some patient care, especially for physiotherapy, dentistry, and occupational health services. Fewer than half of the visits to physicians in 2012 were in municipal health centers, one-third were visits to occupational physicians, and a similar proportion were visits to private physicians (4). Private services are more commonly used by affluent people and occupational health services by people with jobs; as a consequence, these services have differential access according to socioeconomic status (4, 5). Problems in access to health care services in Finland are evident when compared with other Nordic countries. In 2013, 4% of Finns reported that they had not received the treatment they required due to cost, travelling distance, or waiting time. Comparable figures in the other Nordic countries vary between 1 and 2%, except in Iceland, where the proportion is 3.6% (6).

Finland is undergoing extensive social welfare and health care reform (7). The reform is intended to reduce inequities in health and well-being between different population groups, offer equal access to services, and manage rising costs. In order to meet these objectives, social welfare and healthcare services will be combined on all levels. Responsibility for providing healthcare and social services will be assigned to autonomous regions that are larger than the municipalities, but municipalities will still have responsibility for promoting their residents’ health. This will require close cooperation between municipalities and the new autonomous areas.

**Fig. 1:**
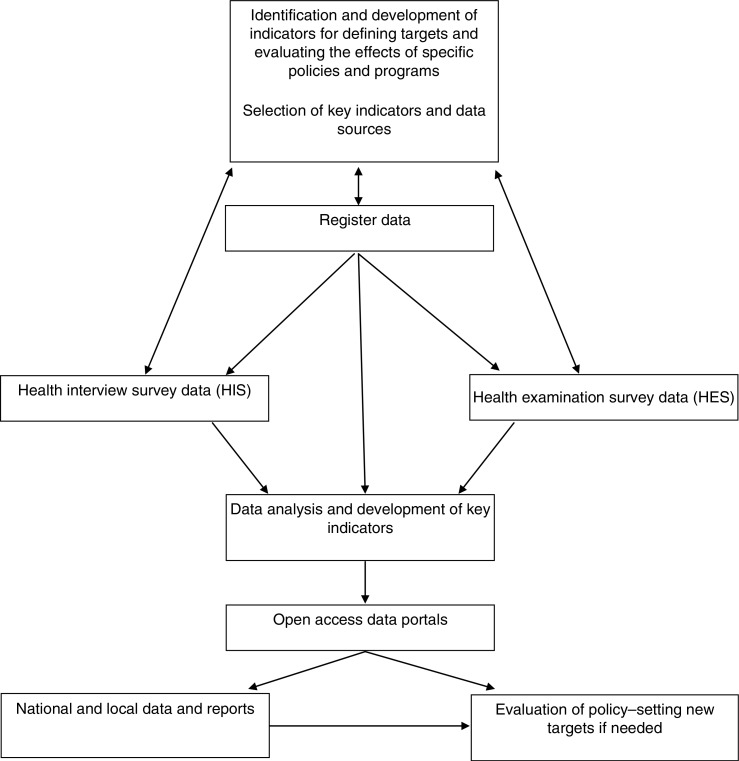
Health monitoring system in Finland.

Health monitoring in Finland is concerned with obtaining information about the health status and health behavior of the population for the purpose of estimating disease burden, identifying populations at highest risk, determining the prevalence of health risks, and evaluating the effects of health policies and interventions. Health monitoring covers several population subgroups based on gender, age group (e.g. youth, adult, and elderly), socioeconomic status, and region. Health monitoring programs are being developed for children, migrants, and ethnic minorities. The health monitoring system in Finland is based on national surveys, national administrative registers, and local patient registers. The process extends from identifying and developing key health indicators and their data sources to the use of data in health monitoring and policy (see Fig. 1). The Finnish key indicators were selected in the National Indicator Project in 2009–2012. The purpose was to ease the use of indicators in local level policy-making by identifying the most important indicators in health monitoring and presenting them in a user-friendly portal (8). Finnish experts have also actively participated in the selection of European Core Health Indicators and the development of the European Health Monitoring System (9, 10). Details of the key national surveys and registers that provide data for health monitoring are given in the following sections.

Well-targeted health promotion actions and welfare management are impossible without comprehensive up-to-date data that help to identify public health problems, strengths and target population groups at the national, regional, and local levels. One of the most important goals of the Finnish health information system is to allow all users free access to data so that they can interpret, share and use this information to take action. At the national level, this information is used in developing and evaluating targeted programs, policies, and legislation, and also in demonstrating progress towards meeting global and or national health targets. At the local level, this information is used for monitoring the health of residents and reporting information to the municipal councils, so that they can better manage and plan health and welfare services and develop and evaluate health promotion activities.

## Health data in Finland

Finland has over 50 years of experience in gathering health survey information about the health and welfare of Finnish adults at both the national and regional levels. Population statistics have been gathered since the 18th century. The data for key health indicators are of good quality and the health information is widely used in health policy-making. Health surveys – both interview and examination studies – are an important source of information on health, welfare, and health services, as well as the determinants of health in populations.

### Registers

Administrative health registers in Finland cover a wide range of data over a person's entire life span (Table 1). The data include information on mortality and cause of death, morbidity (e.g. infectious diseases, cancer, congenital birth defects, heart diseases, occupational diseases, and visual impairments), use of health services (e.g. hospital care, outpatient hospital visits, primary healthcare visits, and cancer screening), and health-related benefits and reimbursements (e.g. disability allowances and reimbursements for medications and private health services). The registers are computerized, and they cover the whole country and all age groups. Unique personal identification numbers enable record linkage across different registers.

**Table 1:** Health data in Finland

Name	Type of data	Year started	Register keeper	Frequency and size	Focus	Target population	Geographical coverage and breakdowns
**Surveys**							
School Health Promotion Survey	HIS (paper and web-based questionnaire)	1996	National Institute for Health and Welfare (THL)	Every second year, *n=*about 200,000	Health, health behavior, and well-being	14- to 20-year-old Finnish adolescent population All pupils in grades 8 and 9 and at first and second years of upper secondary and vocational education	Nation, regions, municipalities
The Regional Health and Wellbeing Study (ATH) *Based on two former surveys, Health Behavior among the Finnish Adult Population and Health Behavior among the Finnish Elderly Population*	HIS (postal and web-based questionnaire)	2012 (previous surveys since 1978)	National Institute for Health and Welfare (THL)	Next survey in 2017; *n*=150,000 in 2012–2014	Health status, health behavior, functional and working capacity, living conditions, well-being, quality of life, perceived need, and use of services	Finnish population, aged over 20 years	Nation, regions, municipalities (with population over 20,000 inhabitants)
Survey on Work and Well-Being among People of Foreign Origin (UTH)	HIS (interview)	2014	National Institute for Health and Welfare (THL) and Statistics Finland	Possibly every 4–8 years; *n*=5,449 in 2014	Employment, education, health, well-being, service utilization	People of foreign origin, aged between 15 and 64 years	Nation, regions
FinHealth *Based on the previous surveys: Mini-Finland, Health 2000, Health 2011, and FINRISK*	HIS/HES (questionnaires and clinical measurements)	2017 (previous surveys since 1972)	National Institute for Health and Welfare (THL)	Every 5 years, *n*=10,000	Health, health behavior (alcohol/drug consumption, physical activity, sleep), functional capacity and working capacity, well-being, living and working conditions	Finnish population aged 25 and over	Nation, regions
Migrant Health and Wellbeing Survey (Maamu)	HES (interview and clinical measurements)	2010–2012	National Institute for Health and Welfare (THL)	Next survey not decided (maybe 2022), *n*=3,000 in 2010–2012	Health, well-being, functional ability	Persons of Russian, Somali, and Kurdish origin	Six cities
**Registers**							
Morbidity							
Cancer Register	Register	1953	National Institute for Health and Welfare (THL)	Every year	Data on cancer cases and deaths	Finnish population	Nation, regions, municipalities
Register of Occupational Diseases	Register	1964	Finnish Institute of Occupational Health (FIOH)	Every year	Data on occupational diseases	Finnish population	Nation, regions, municipalities
Cause of Death Register	Register	1969	Statistics Finland	Every year	Data on deaths and causes of death	Finnish population	Nation, regions, municipalities
Reproductive health							
Medical Birth Register, Registers on Congenital Malformations, Register of Induced Abortions and Sterilizations	Register	1987/1963/1977	National Institute for Health and Welfare (THL)	Every year	Data on parturients, their newborn babies, and care during pregnancy, childbirth, and early neonatal period (since 1987). Live births and stillbirths with congenital anomaly and termination of pregnancy due to congenital anomalies (since 1963). Legally induced abortions and sterilizations (since 1977)	Finnish population	Nation, regions, municipalities
Drugs and addictions							
Register of Adverse Drug Reactions	Register	1966	Finnish Medicines Agency (Fimea)	Every year	Data on adverse drug reactions	Finnish population	Nation, regions, municipalities
Drug Surveillance Register	Register	1982	Finnish Medicines Agency (Fimea)	Every year	Data on prescriptions for medicinal preparations classified as narcotics	Finnish population	Nation, regions, municipalities
Infectious diseases							
Register of Infectious Diseases	Register	1989	National Institute for Health and Welfare (THL)	Every year	Data on infectious diseases	Finnish population	Nation, regions, municipalities
Environment and health							
Register of Persons Exposed to Cancer-Hazardous Material	Register	1979	Finnish Institute of Occupational Health (FIOH)	Every year	Data on persons exposed to carcinogenic material	Finnish population	Nation, regions, municipalities
Health and social services							
Register of primary healthcare visits Hospital Discharge Register (healthcare institutions) Discharge Register (social institutions)	Register	2011/1967/1994	National Institute for Health and Welfare (THL)	Continuous	Data for all patient encounters within the publicly provided primary care (primary healthcare centers), hospital inpatient care (since 1967), surgical procedures (since 1994), hospital outpatient care (since 1998), inpatient care in social institutions and home help (since 1994)	Finnish population	Nation, regions, municipalities
Mass Screening Register (cervical and breast cancer)	Register	1968/1987	National Institute for Health and Welfare (THL)	Every year	Data on breast and cervical cancer screening	Finnish population	Nation, regions, municipalities
Child Welfare Register	Register	1991	National Institute for Health and Welfare	Every year	Data on children taken into custody	Finnish population	Nation, regions, municipalities
Registers on Orthopedic Endoprostheses Register of Dental Implants	Register	1980/1994	National Institute for Health and Welfare (THL)	Every year	Data on orthopedic endoprostheses (since 1980) and dental implants (since 1994)	Finnish population	Nation, regions, municipalities
Central register on Health Care Personnel	Register	1955	National Supervisory Authority for Welfare and Health (VALVIRA)	Every year	Data on all licensed health care personnel	Finnish population	Nation, regions, municipalities
Social protection							
Register of Social Assistance	Register	1985	National Institute for Health and Welfare (THL)	Every year	Data on social assistance	Finnish population	Nation, regions, municipalities
Register of Pensions	Register	1962	Finnish Centre for Pensions (FCP)	Every year	Data on pensions	Finnish population	Nation, regions, municipalities
Registers on social benefits under the National Sickness Insurance	Register	1964	Social Insurance Institute (KELA)	Every year	Data on drug reimbursements, sickness leave, rehabilitation, and other health- and social welfare–related allowances and benefits	Finnish population	Nation, regions, municipalities

HIS, Health Interview Survey; HES, Health Examination Survey.

Despite the large volume and variety of information collected in registers, surveys are also needed. They supplement information in the population registers. Registers seldom contain information on health behavior, self-perceived health, citizens’ own experiences and attitudes, or functioning. Among Finnish registers, only the Medical Birth Register collects data on maternal smoking during pregnancy, and only the Primary Health Care Register collects data on smoking, weight, and height. Further, although registers give information on the use of services, they are not good at describing existing or future needs for services and how these needs are met (or not). More importantly, registers seldom gather information on health determinants such as housing and income. However, comprehensive monitoring of health by population subgroups, especially by socioeconomic status, can be done with linkages to other registers. Due to European data protection regulations and their national interpretation, there are limitations to operationalizing all possible linkages (11). Currently, national data protection legislation supports the collection and use of personal data on health, without the consent of the registered people, for research and statistical purposes. However, such linkages are undertaken on an *ad hoc* basis only. Informed consent is, needed if data are collected directly from the citizens, for example, questionnaires or biological samples, and further linked to register information.

### Surveys

Both Health Examination Surveys (HESs) and Health Interview Surveys (HISs) are carried out at regular intervals in Finland (Table 1).

HISs provide information on self-reported health behaviors, health status, diseases, health service needs, and service utilization. They also provide information on opinions and attitudes. HISs in Finland are mainly carried out by post (and increasingly also by the Internet) through written questionnaires but also by telephone or face-to-face interviews. The two main surveys providing both regionally and nationally representative data are the Regional Health and Wellbeing Study (ATH) and the School Health Promotion Study (Table 1). Some previous HISs in Finland have already been merged or will be merged in the future with the ATH survey. The 2014 European Health Interview Survey in Finland was carried linked to the ATH survey.

The HISs may suffer from reporting bias because respondents may be unaware of their health problems (e.g. risk factors) or they may underreport or overreport based on their interpretation of social desirability. Examples of such indicators include blood pressure, blood cholesterol levels, and even body height and weight, as well as some mental health problems. These indicators can be measured reliably only by HESs, which provide information that cannot be obtained objectively or at all from other sources. HESs always include questionnaire-based data, but physical measurements and biological sample collection are also incorporated into these surveys. Only a few countries in Europe have a system of repeated national HESs (Finland, England and Scotland, Germany, Ireland, the Netherlands, and Poland) (12).

Two national HES traditions, the FINRISK Survey and the Health 2000 and Health 2011 Surveys, will be merged into one survey, the FinHealth Survey, in 2017 (Table 1). The new combined survey, which will contain relevant up-to-date questionnaire items and health examination measurements from the previous surveys, will be implemented according to European standards (12). The broad aim of this initiative is not only to reduce the number of different surveys and to save costs but also to pay attention to data quality and address decreasing participation rates. Another aim is to develop surveys with comparable methods at the national level rather than separate surveys carried out at regional or local levels. It has been acknowledged that it is not effective to conduct numerous small surveys, for reasons of cost and also due to difficulties in collecting valid and reliable data. The aim is to build expertise in survey methods at the national level, as well as to develop better coordination for the national health survey system.

National surveys in Finland have provided data for identifying and monitoring gender-based, socioeconomic, and regional differences in health status, health behavior, and health service utilization. However, as these routine surveys cannot be used to monitor the migrant population in Finland, two specific studies have been developed: the Migrant Health and Wellbeing Survey (Maamu), a HES conducted in 2010–2012, and the Survey on Work and Well-Being among People of Foreign Origin, an HIS conducted in 2014. Both surveys allow comparisons with the general population by utilizing the data from the Health 2011 survey and the ATH surveys.

## Health monitoring and health policy evaluation

Both survey- and register-based data are valuable tools for evaluating health policy both at national and local levels in Finland.

National surveys have shown progress in, for example, reduction in smoking and many other chronic disease risk factors (13). They also make it possible to identify increases in the prevalence of depressive disorders (14). Significant differences between migrant groups and the general population have also been reported through these surveys (15). However, both register-based studies and surveys have shown that some health policy targets have not been met, and socioeconomic differences have remained large in regard to health status, health service utilization, and mortality, compared to other European countries (16–20).

The municipalities use local information, in drawing up their statutory welfare reports (Section 12 of the Finnish Health Care Act), which are provided to the municipal councils. A comprehensive, statistics-based account must be provided once during a council's 4-year term and a concise report is required annually. The municipalities are obligated to monitor and report their residents’ health by population subgroups. The welfare report is the instrument that leads the planning, monitoring, evaluation, and management of the welfare policy within the municipality. It is intended that welfare reports help municipalities prevent health problems, decrease the need for services, and minimize unfair inequalities between citizens. Welfare reports should be prepared in collaboration with other municipal sectors (environment, technical, culture, sport, and leisure time) and the data should be integrated with national policy goals. Some municipalities also disaggregate their data by residents’ education, which can inform the allocation of specific equity-orientated measures. However, if the focus is only on the most disadvantaged groups, other public health issues may not be tackled sufficiently. Therefore, actions must be universal, but with a scale and intensity that is proportionate to the level of the disadvantage (21). In addition to welfare reports, since 2011 the Finnish legislation has also obligated municipalities to utilize HIA. However by 2015, only a third of municipalities reported using HIA in their work (22).

## Dissemination of health and welfare data

There are various web-based portals with key indicators that support the municipalities in Finland in regard to health monitoring. The portals provide register- and survey-based data, and the information is updated annually. The indicators are presented at the national, regional, and local levels.

*The Sotkanet.fi* (www.sotkanet.fi) database includes mainly register-based data and provides national and local data on demographic variables, healthcare provision, and living conditions, as well as life expectancy, mortality, and cancer incidence. Many indicators are available by municipality and region as well as over time, from 1990. Data are presented by gender and age, but not by socioeconomic status. The service is available in Finnish, Swedish, and English.

*Our Health* (www.thl.fi/terveytemme) is an online service that offers an interactive web-based tool for the visualization of health and well-being indicators based on survey data. The data, including the confidence intervals, are presented in maps, charts, and tables. The aim is to facilitate the statutory monitoring of population health and well-being as well as enabling easy identification of differences between socioeconomic groups. The service is currently available only in Finnish.

**Fig. 2:**
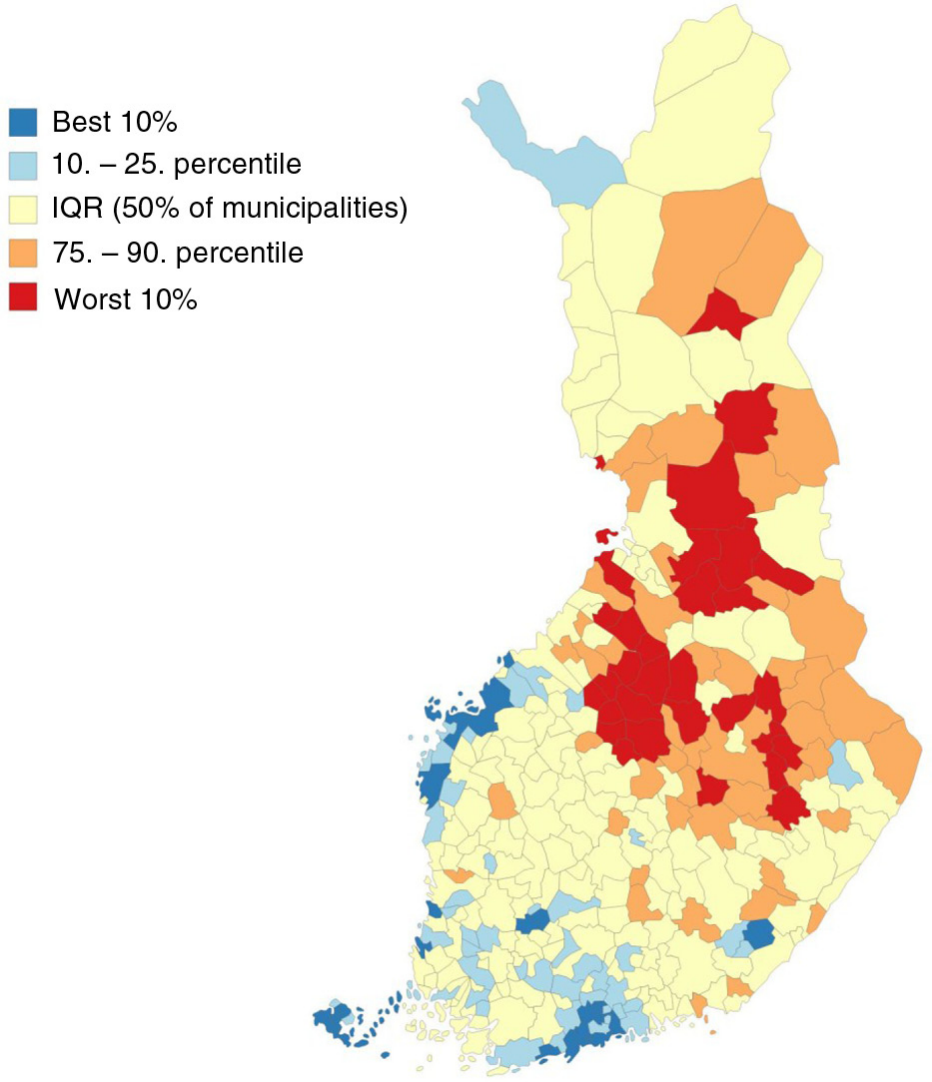
Morbidity index of Finnish National Institute of Health and Welfare 2010–2012, age-standardized. Data source: www.thl.fi/sairastavuusindeksi.

As an example of the results of Our Health online service, Fig. 2 shows that people in the southern and western parts of Finland are healthier in many ways, compared with those living in the eastern or northern parts of Finland.

*Welfare Compass* (www.thl.fi/hyvinvointikompassi) is an online service that provides a collection of key indicators and gives an overview of the development of welfare, health, and social and healthcare services in Finland. It allows easy visualization for comparing municipalities, hospital districts, or regions. The service is available both in Finnish and English.

*TEAviisari* (www.thl.fi/teaviisari) is an online service that depicts municipalities’ activities in promoting their inhabitants’ health. The service supports the planning and management of municipal and local health promotion. The service is available in Finnish and English.

*Palveluvaaka* (www.palveluvaaka.fi/) is an online service where the citizens can search, compare, and evaluate social welfare and healthcare services. The service is available in Finnish and Swedish.

In addition to these portals, further means of data dissemination are needed in Finland. Linkage of data from different registers and surveys is needed to show key health indicators by, for example, socioeconomic status and ethnic background in order to promote equity in health. Moreover, health monitoring needs to cover all age groups, including children. Although child and school health clinics provide regular health examinations with good coverage to all Finnish children, the information gathered in the administrative registers is limited. New systems are under development to collect comparable data from child health clinics.

## Key lessons learned from Finnish experiences

In Finland, the availability of data on key health and social indicators is good. Health information is widely used in evaluating how national health policy targets have been met in different parts of the country and in different population groups.

Both register-based studies and surveys have shown progress in addressing chronic disease risk factors such as smoking. However, some health policy targets have not been met and socioeconomic and regional health inequalities are large (Fig. 2). It is important that political decision-makers and healthcare professionals facilitate wider and more comprehensive use of available information.

The social welfare and healthcare reforms in Finland aim to achieve equal access to services and lower costs. In order to evaluate the success of these reforms, it is necessary to use local information to identify key indicators. Moreover, because the private sector is likely to become more involved in service provision, monitoring inequalities in health and welfare will be even more important to ensure that the reforms are not widening gaps between population groups. However, the key decisions on how health and welfare will be monitored under the new system will not be made before 2017–2018.

Finland is progressive in this area. In many other countries, there is continued reliance on traditional mortality statistics for health planning, and data on other health outcomes are crude or non-existent (10). The Finnish model, where different data sources are widely used for health monitoring purposes, can provide the initiative for improving health information systems in other countries. Moreover, the European Health Interview Survey (EHIS) and Health Examination Survey (EHES) can offer a means of improving the availability of useful data on key health indicators in many European countries in the future.
